# Melatonin: Countering Chaotic Time Cues

**DOI:** 10.3389/fendo.2019.00391

**Published:** 2019-07-16

**Authors:** Josephine Arendt

**Affiliations:** Faculty of Health and Medical Sciences, University of Surrey, Guildford, United Kingdom

**Keywords:** melatonin, circadian, seasonal, health, sleep, light, desynchrony

## Abstract

Last year melatonin was 60 years old, or at least its discovery was 60 years ago. The molecule itself may well be almost as old as life itself. So it is time to take yet another perspective on our understanding of its functions, effects and clinical uses. This is not a formal review—there is already a multitude of systematic reviews, narrative reviews, meta-analyses and even reviews of reviews. In view of the extraordinary variety of effects attributed to melatonin in the last 25 years, it is more of an attempt to sort out some areas where a consensus opinion exists, and where placebo controlled, randomized, clinical trials have confirmed early observations on therapeutic uses. The current upsurge of concern about the multiple health problems associated with disturbed circadian rhythms has generated interest in related therapeutic interventions, of which melatonin is one. The present text will consider the physiological role of endogenous melatonin, and the mostly pharmacological effects of exogenous treatment, on the assumption that normal circulating concentrations represent endogenous pineal production. It will concentrate mainly on the most researched, and accepted area of therapeutic use and potential use of melatonin—its undoubted ability to realign circadian rhythms and sleep—since this is the author's bias. It will touch briefly upon some other systems with prominent rhythmic attributes including certain cancers, the cardiovascular system, the entero-insular axis and metabolism together with the use of melatonin to assess circadian status. Many of the ills of the developed world relate to deranged rhythms—and everything is rhythmic unless proved otherwise.

## Background

The essential physiological role of the pineal hormone melatonin is to provide information on photoperiod (day length) for the organization of seasonal physiology (1). It does not appear to have an essential function in the circadian system but has clear modulatory effects. These functions depend primarily upon G-protein linked membrane receptors MT1 and MT2 (2, 3). It may also be concerned with periodicities other than circadian and seasonal but as yet this is an emerging field (4–6). Its profile of secretion reflects the length of the scotoperiod (night). Even humans with ubiquitious artificial light, can show this change in suitable conditions (7). It was originally called a photo-neuroendocrine transducer molecule and subsequently, informally, a darkness hormone. Most if not all vertebrate photoperiodic species depend on this signal to time seasonal breeding (1), but see (8).

Melatonin is synthesized from tryptophan via 5-hydroxytryptophan and 5-hydroxytryptamine (serotonin). Then *N-*acetylation of serotonin by *N*-acetyl transferase (arylalkylamine N-acetyl transferase, AA-NAT) to *N*-acetylserotonin (NAT) and *O*-methylation by acetylserotonin O-methyltransferase (ASMT), [previously known as hydroxyindole-*O*-methyltransferase (HIOMT)] to melatonin (*N*-acetyl-5-methoxytryptamine). A major increase (7–150 fold) in the activity of AA-NAT at night is usually rate limiting in melatonin production. The rhythm of production is endogenous, being generated by clock genes in the suprachiasmatic nuclei (SCN), the major central rhythm-generating system or “clock” in mammals (9, 10). The rhythm, as for the circadian system in general, is synchronized to 24 h primarily by the light-dark cycle acting via the retina and the retinohypothalamic projection to the SCN (9).

Light of suitable intensity and spectral composition can phase shift and entrain circadian rhythms. Light also suppresses melatonin production at night (11). The amount of light required for suppression varies from species to species, with time of night, with spectral composition and with previous light exposure. In humans,~2,000 lux full spectrum light (domestic light is around 50 to 500 lux) is required for complete suppression at night. However, much lower intensities will partially suppress and shift the rhythm (12, 13). A non-image forming photoreceptor system of light sensitive retinal ganglion cells is implicated in these effects, with a pivotal role of the photopigment melanopsin, although in normal circumstances input from rods and cones is also used (14, 15). In humans maximum suppression and phase shifting for equal numbers of photons is given by blue light (460–480 nm).

In humans melatonin is metabolized, ~70% to 6-sulphatoxy melatonin (aMT6s), primarily within the liver, by 6-hydroxylation, followed by sulfate conjugation (with some species variaions). A number of minor metabolites are also formed, including the glucuronide conjugate. N1-acetyl-N2-formyl-5-methoxykynuramine and N1-acetyl-5-methoxy-kynuramine, were initially reported as brain metabolites (1, 16) but have proved difficult to detect in plasma or urine except after administration of exogenous melatonin (17). Exogenous oral fast release or intravenous melatonin has a short metabolic half-life (20 to 60 min, depending on author and species), with a large hepatic first pass effect and a biphasic elimination pattern (18). Slow release/prolonged release/surge sustained preparations are of course designed to extend the time of high circulating melatonin [e.g., (19)]. It has low bioavailability in general although transmucosal administration increases bioavailability (20). A critical feature of exogenous melatonin with regard to its clinical uses is its very low toxicity and lack of addictive properties (21, 22).

### Source of Endogenous Melatonin

The pineal gland is the source of the vast majority of circulating melatonin in mammals [e.g., (23–25)]. Its synthesis and presence has been described in a large number of other structures, but they do not appear to contribute significantly to blood levels in, for example, humans and rodents, except following specific manipulations of synthesis such as provision of excess precursor (26, 27). Pinealectomy leads to loss of the rhythm and usually undetectable amounts of circulating melatonin in mammals although with high sensitivity assays traces may be found. This is curious in view of reported non-pineal melatonin synthesis, sometimes in very large amounts, and the highly lipophilic/amphipathic nature of the molecule which penetrates all compartments rapidly (28, 29). Superior cervical ganglionectomy (denervation of the pineal) also abolishes the rhythm (30). Retinal melatonin is of major interest [e.g., (31, 32)] but beyond the scope of this text.

Non-pineal melatonin has been considered to act locally (29). Local effects have been invoked with regard to metabolism, immune function, gut function, inflammation, membrane fluidity, mitochondrial function, apoptosis (both stimulation and inhibition), free radical scavenging, direct anti-oxidant activity, influence on anti-oxidant enzymes, redox status, radioprotection, and others (33). Protective therapeutic effects are reported with regard to many various systems but notably neural, oncological and cardiovascular. Some of these effects are thought not to require receptor signaling, although melatonin receptors are now found widely distributed.

Very recently it has been demonstrated that in the mouse brain melatonin is exclusively synthesized in the mitochondrial matrix. It is released to the cytoplasm, thereby activating a mitochondrial MT_1_ signal-transduction pathway which inhibits stress-mediated cytochrome *c* release and caspase activation: these are preludes to cell death and inflammation. This is a new mechanism whereby locally synthesized melatonin protects against neurodegeneration. It is referred to as automitocrine signaling (34). Another recent addition to our understanding is the observation that a gut bacterium, *Enterobacter aerogenes*, expresses an endogenous circadian clock that is responsive to signals from the host's circadian system, the hormone melatonin, and changes in temperature. This establishes a prospective link between melatonin as a peripheral circadian zeitgeber (time cue), and the gut (35).

Peripherally administered exogenous melatonin (sometimes in very high pharmacological doses) can presumably access the various structures involved in local effects even though the non-pineal endogenously synthesized melatonin does not apparently get out. The gut is reported to contain several 100 fold more melatonin than the pineal gland, but does not contribute to the circulating rhythm of melatonin. It is said to sustain the (very low) day time plasma levels (27). Although melatonin is present in some foodstuffs (36), in the authors experience it is hard to show an increase in plasma melatonin after a normal meal. This area has been extensively reviewed by others and will also feature in this volume.

In principle the established role of melatonin in rhythmic function is not necessarily incompatible with the use of high doses for ‘protective’ effects. Unless desensitization of the melatonin membrane receptors occurs as a result of continuous high circulating concentrations (37, 38) and compromises functions responsive to low levels of melatonin such as sleep and circadian phase.

### Melatonin Physiology

#### Seasonal Rhythms

A truly distinct physiological role for melatonin was initially indicated by the fact that pinealectomy or ganglionectomy (which abolished the rhythm of circulating melatonin) abolished the ability of photoperiodic mammals to time seasonal physiology according to the day length (with very rare exceptions) (39, 40). Melatonin secretion, long in long nights, short in short nights provided the information, via photoneurotransduction, to body physiology for the organization and timing of seasonally rhythmic functions such as reproduction and coat growth. Replacement of the endogenous melatonin signal by long or short profiles of exogenous melatonin at the same plasma concentration as the endogenous signal was equipotent with day length for control of seasonal timing. The downstream events have now been investigated in considerable detail [e.g., (41, 42)] and melatonin treatment to shift the timing of seasonal breeding in domestic species such as sheep, mink, and goats to maximize profit is now commercialized (43).

Humans have residual seasonality as evidenced by numerous physiological variables. and particularly by the existence of seasonal affective disorder and its treatment by suitable light exposure (and on occasion by melatonin as a chronobiotic) (44, 45). So for the therapeutic use of melatonin in humans it should never be forgotten that this hormone has profound effects on animal seasonal functions. The evidence for anti-gonadotrophic effects of high amounts in humans is quite substantial (46–49), and an influence on pubertal development is possible but not demonstrated. For example photoperiod, via melatonin profile, times puberty in sheep (50), melatonin inhibits LHRH stimulation of LH in the neonatal rat pituitary (46, 51), and in a case report of successful treatment of delayed puberty in a young woman, her production of melatonin declined dramatically (52). A serious attempt to develop it as a contraceptive was made some 25 years ago (53).

#### Circadian Rhythms

By contrast it is quite difficult to show major effects of pinealectomy on circadian rhythms and even on sleep. Initial investigations on pinealectomized rats showed little effect on activity rest cycles (54). These have been reinforced by a very recent study, again indicating that removal of the pineal has no effect on rodent sleep (55). However, if animals were subjected to an abrupt phase shift (as in jet lag), they adapted faster to the new schedule without a pineal gland (56, 57). This suggested that the pineal, and by inference melatonin, acted as a brake on abrupt changes of phase, these being undesirable in a natural environment. This possibility is reinforced by the fact that suppression of melatonin production by the beta blocker atenolol leads to faster adaptation to light-induced phase-shifts in humans (58). Ironically therefore whilst exogenous melatonin is used to hasten adaptation, it is possible that a function of endogenous production is to do the opposite.

**Figure 1:**
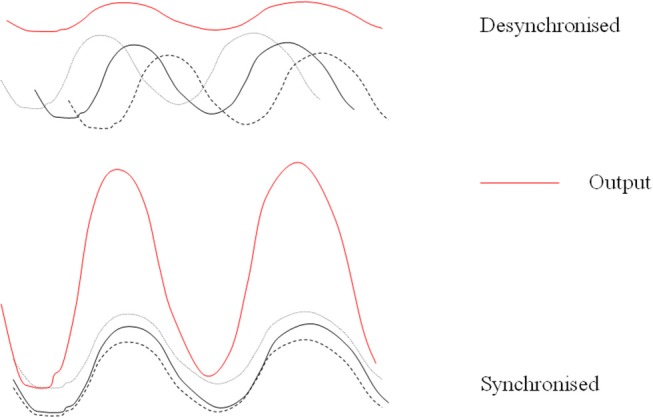
Desynchronized rhythms lead to lowered output of a multi-oscillatory system. Simplified diagram of how melatonin might act endogenously to maintain coupling and synchronization of its target outputs and how desynchronized rhythms may lead to lowered production of melatonin itself.

Further support for a modulatory role in the circadian system is evident from the fact that in constant bright light pinealectomized animals, in comparison to sham operated animals, show more disrupted rhythms in wheel running, general activity, body temperature, and heart rate (54). Several authors have suggested that a function of the pineal and melatonin is to act as a coupling agent with regard to rhythmic systems (“circadian glue”). This would fit with the suggested role of maintaining the status quo (Figure 1). It could also be considered with respect to any effects on other periodicities.

Since melatonin is constantly referred to as the sleep hormone in the media, it is worth stating that it is not essential to sleep although we sleep better when in phase with rhythmic circulating melatonin and the rest of the circadian system (59). It is, to say the least, difficult to study pinealectomized humans before and after pinealectomy. However, this has been done prospectively with pre and post-operative polysomnography, the so-called gold standard for sleep measures. No effects of the missing pineal on sleep were seen (60). This was a small but careful and exceedingly rare study, it merits serious attention. The final comment on melatonin as a “sleep hormone” is that it most certainly is not so in nocturnal rodents—it is a darkness hormone not a sleep hormone.

## Countering Changes in Time Cues

The accumulated knowledge on the deleterious effects of abruptly changing time cues in for example shift work and jet lag [e.g., (61–67)] lead to the suggestion that one function of endogenous melatonin is to protect against abrupt short term changes of phase by maintenance of the circadian status quo.

### Effects on Circadian Rhythms

**Figure 2:**
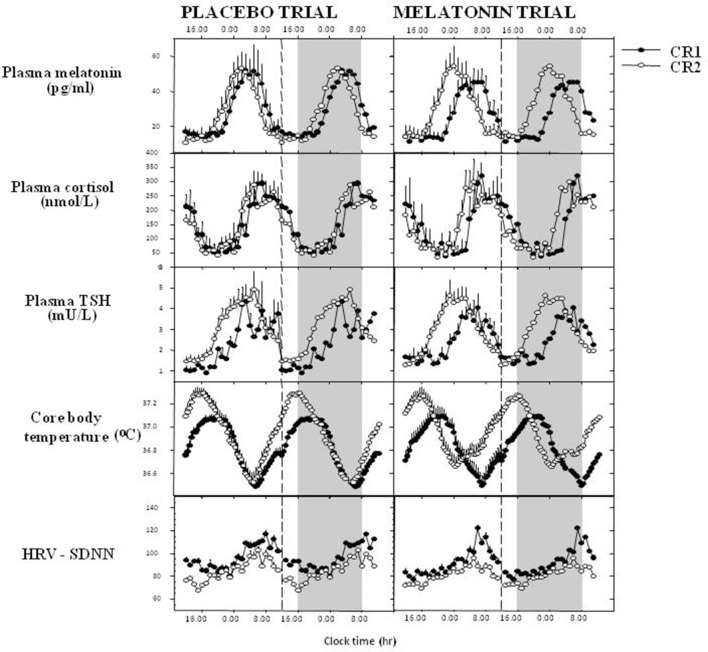
Melatonin phase shifts all measured rhythms in humans. 1.5 mg surge sustained release at 1600 h daily for 8 days, recumbent, <5 lux, 1600–0800 h, evaluated in constant routine. Data derived and redrawn from Rajaratnam et al. (19), Middleton et al. (69), and Vandewalle et al. (71). CR1, 1st constant routine; CR2, 2nd constant routine; TSH, thyroid-stimulating hormone; HRV-SDNN, heart rate variability- standard deviation of the interbeat interval of normal sinus beats.

Early work indicated that timed exogenous melatonin treatment, pharmacological in rats, close to, but still usually supra-physiological in humans, could entrain activity rest cycles in rats, shift circadian phase, assessed using endogenous melatonin as a marker rhythm, and synchronize free-running rhythms in humans. For references see (68).The most obvious manifestation in humans is the timing of the sleep-wake cycle. Phase shifts and entrainment after timed low dose melatonin treatment were evident initially in the rhythm of sleep and of melatonin itself and then in the timing of all the circadian rhythms observed (19, 69, 70) (Figure 2). Both phase advances and phase delays can be induced dependent on the time of treatment (72) which can be expressed as a Phase Response Curve or PRC. The melatonin PRC is approximately the opposite of that to light pulses and for maximum effect the two treatments can be combined with careful timing (73, 74). If the period of melatonin secretion is considered to be “biological night” then low dose (0.5–5 mg) treatment in the late afternoon will advance circadian phase and sleep (75), whereas treatment in the early 'biological morning' will cause phase delays. Thus, it is important to know or predict circadian phase in order to time treatment correctly. The clearest demonstration of entrainment could be seen in free-running blind subjects (76, 77) and in sighted subjects kept in a time free environment (78).

Melatonin clearly has both a direct sleep inducing effect coupled with a circadian phase shift (70). Importantly it was shown to act directly at the level of the suprachiasmatic nucleus (79) to modify its rhythmic activity, amplitude and phase, via G-protein linked membrane receptors now extensively characterized as MT1 and MT2 (MTR1A/Mel1a, Mel1b/MTR1B) (2, 3, 79–81). MT1 was associated with suppression of SCN electrical activity and MT2 with phase shifts, with some redundancy and cooperation between these subtypes. However, a recent report describes a lack of overlap in mouse brain structures showing one or other of these receptors. The authors state that “the expression and distribution of MT2 receptors are much more widespread than previously thought, and there is virtually no correspondence between MT1 and MT2 cellular expression” (82). A third receptor MT1c is not found in mammals but a related G-protein coupled receptor GPR50 has 45% identity in amino-acid sequence with MT1 and MT2 and is thought to be the ortholog of Mel1c in mammals (83). It may have a role in glucocorticoid receptor signaling with implications for peripheral control of circadian rhythms (84). Melatonin may also directly affect clock genes in the SCN [e.g., (85)].

Others will report in this volume in detail concerning receptors and downstream signaling events. It is just noted here that a recently reported signaling by melatonin receptors in the SCN appears to require G-protein-coupled inwardly rectifying potassium (GIRK**)** channels: a widely distributed physiological neural communication system (86). The authors propose this as some of the explanation for the variety of reported effects of this hormone in mood and other neurological disorders.

In addition to effects on the central rhythm generating system, melatonin also influences peripheral oscillators for example in the pars tuberalis, the cardiovascular system, the skin, the adrenal (87–90), various primate fetal tissues (91) and possibly the expression of sirtuin 1 (a histone deacetylase) which is thought to enhance circadian amplitudes and may prolong survival (92). Melatonin clearly has the potential to influence all rhythmic function by virtue of its universal distribution, however very recent data indicates a major role for glucocorticoids in entraining/synchronizing peripheral clocks (84). It is clear that melatonin can manipulate the circadian system. It may well be that a combination of melatonin, light and glucocorticoids could provide the most efficient realignment of both centrally generated and peripheral clocks.

### Circadian Desynchrony

In a natural environment, changes in circadian rhythms occur due to seasonal influences, notably changes in photoperiod leading to shorter or longer melatonin secretion profiles (93). In humans a seasonal effect is more commonly seen as delayed rhythms in winter (94) especially in polar regions with no sunlight for long periods of the year (95). A duration change in melatonin profiles is rarely seen in humans in temperate zones but can be elicited with artificial light/darkness (7). one explanation is that the onset of secretion is delayed in winter, but the subject is required to get up to work in the morning. Thus, the full expression of the profile is curtailed by artificial light suppression both in the evening and in the morning. In the urban environment of today artificial light is everywhere leading to changes in sleep. It is interesting to compare sleep in similar rural communities with and without artificial light. Artificial light clearly impacts the timing and duration of sleep (96).

The abrupt changes in the light dark cycle and consequent desynchrony experienced by shift workers and time zone travelers are now known to be associated with increased risk of accidents, sleep deficits, lowered alertness and performance, gut problems, lowered fertility, perhaps psychiatric problems, and increased risk of major disease such as cancer, diabetes, metabolic syndrome and heart disease (67, 97).

The central pacemaker of the SCN adapts slowly to these abrupt changes and peripheral oscillators adapt at different rates, such that the body is in a state of both internal and external desynchrony (63, 65, 98–103). Melatonin (and other rhythms being driven by the SCN) is slow to adapt, endeavoring to maintain the circadian status quo. It is out of phase during adaptation and may be partly suppressed by sufficient light at night (LAN), e.g., in shift workers, although not all studies concur. This is thought to be a causal factor in the increased risk of, for example, breast cancer by some authors and will be discussed later (97, 104, 105). However, the entire circadian system is disturbed in these circumstances, not just melatonin.

Out of phase rhythms with or without suppressed melatonin may well be involved in the deleterious effects of shift work (63, 104, 106, 107). As yet there is no definitive linkage between a particular degree of melatonin suppression and any deleterious effects. Some people appear to lead perfectly normal lives with very low or even undetectable melatonin. But there is no long term information on disease risk in these low melatonin secretors. Certainly desynchrony will be one cause of disordered sleep, since we sleep better when in an appropriate phase relationship with the melatonin rhythm. But when the entire circadian system is dysregulated numerous other effects and potential causes can be invoked.

## Sleep

**Figure 3:**
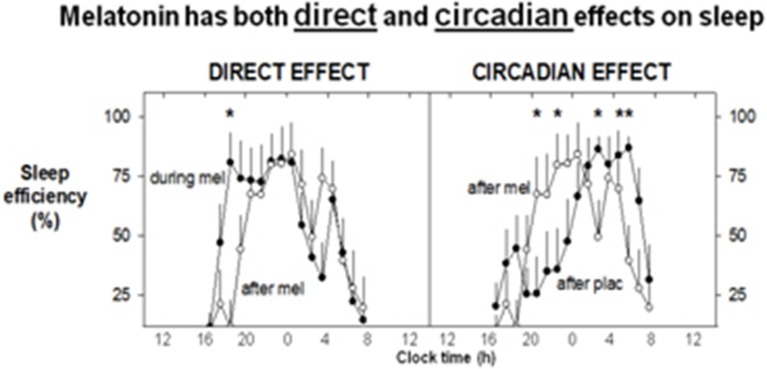
Exogenous melatonin has both direct and indirect effects on sleep. 1.5 mg surge sustained release at 1600 h daily for 8 days, recumbent, < 5 lux, 1600–0800 h, evaluated in constant routine. Mean sleep efficiency levels (% per hour: *n* = 8). The direct, sleep-facilitating effect of melatonin **(left)** is illustrated by a comparison between sleep efficiency profiles on the last day of melatonin treatment and sleep efficiency on the following washout day. Increased sleep efficiency (direct effect) is observed for the first 2–3 h during melatonin treatment. The circadian effect of melatonin on sleep **(right)** is shown by comparing the sleep efficiency on the washout day (the day after melatonin or placebo). On the washout day, placebo was administered to all participants. A shift in the distribution of sleep can be observed after melatonin treatment, with the major bout of sleep occurring earlier in the sleep opportunity. On the corresponding day after placebo, the major bout of sleep occurred later in the sleep opportunity, although an initial rise in sleep efficiency is noted at around the commencement of the sleep opportunity. With Permission from Rajaratnam et al. (70). *Significant difference between CR1 and CR2.

Melatonin itself and its agonists have been developed primarily to treat sleep disturbance (108) but with currently expanding possibilities for clinical therapeutics. Its immediate effects on sleep were initially investigated long ago, first by Aaron Lerner who identified melatonin. Its immediate effects on sleepiness/sleep are accompanied by a dose-dependent lowering of core body temperature in near physiological doses (75, 109), and this has been invoked as part of the mechanism of action. This phenomenon has been linked to sleep induction in a series of careful trials (110, 111). Melatonin has clear, time-dependent direct (soporific) and phase shifting effects on human sleep in near physiological/low pharmacological doses (70, 112) (see Figure 3). It's rhythm is closely associated with the timing of sleep and sleep propensity, and inversely with that of core temperature (113).

When treatment with melatonin is related to the so-called circadian rhythm sleep disorders (CRSDs) it is a logical development exploiting both the direct and phase shifting properties (68). CRSDs include delayed sleep phase, advanced sleep phase, free-running sleep, and the sleep detriments of jet lag and shift work. Although the endogenous central pacemaker has a major role in timing sleep, humans exercise choice according to desire or necessity, as to when they try to sleep. This means that sleep rhythms are not a pure manifestation of the circadian system. True circadian phase shift and/or entrainment requires a demonstration that a marker rhythm such as melatonin, cortisol or core temperature is entrained. If treatment is timed to maximize the phase shifting and direct sleep inducing effects of melatonin it can be very successful, particularly with respect to mistimed sleep.

### Delayed Sleep Phase Syndrome (DSPS)

Typically a subject reports inability to sleep before 2 to 6 a.m. When not required to maintain their schedule—i.e., weekends, holidays, etc.—they sleep without difficulty, and will awaken spontaneously after a sleep period of normal length. Severe cases of DSPS are relatively common in adults (114).The incidence of clinically important DSPS in students/adolescents may well be as high as 7%. An early meta-analysis (115) concluded that there was no evidence for efficiency of melatonin in treating secondary sleep disorders and sleep disorders associated with sleep restriction. Sadly they did not specifically select publications which gave treatment at the correct time but they did conclude that DSPS was an area where melatonin could be useful. The British Medical Journal published some “rapid responses” to this publication which were highly critical of the summary and conclusions.

Some very careful work has been carried out in adults and children with DSPS, measuring circadian phase and timing treatment to 5 h before melatonin onset for maximum phase advance. Sleep timing was advanced, criteria of general health were improved, there were no later effects on reproductive health and in some cases treatment could eventually be withdrawn. A meta-analysis (116) describes the quality studies in both children and adults published up to 2010 and concludes that melatonin treatment induced an earlier phase (melatonin onset, 1.18 h) reduced sleep latency of 23 min and earlier clock onset of sleep by 0.67 h. Timed melatonin treatment was recommended for DSPS by the American Academy of Sleep Medicine (117). More recent well-controlled trials have strongly supported the use of timed melatonin with or without timed light exposure for DSPS (118, 119). However, by no means all diagnosed DSPS patients have a circadian delay as well as a sleep delay (120).

For Advanced sleep phase syndrome (ASPS), there is little information on melatonin treatment.

### Shift Work

Another common situation with temporarily displaced sleep is that of shift work. There is sparse evidence that melatonin can help day time sleep during real life night shift and night time sleep after return to day work, although anecdotally melatonin is used. An early real life study reported greater day sleep duration after night shift when subjects left work early (6 am, before conflicting bright light) and took melatonin (5 mg) before day sleep (121). A later real life timed study addressed both day and night time sleep and was successful in its carefully timed use of melatonin (3 mg) 1 h before bedtime (122). An increase in sleep duration of 15–20 min was obtained and a reduction in sleepiness at work (subjective measures). In a series of simulation shift work studies Eastman and colleagues have clearly shown that timed melatonin (1.8 mg sustained release) and bright light exposure will partially shift phase, such that day sleep is improved when working nights and subjects rapidly readapt on return to day work (73, 123, 124). Data from real life shift work studies were positive in one review (125) and the American Academy of Sleep Medicine approves its use in shift work sleep disorder (117).

### Jet Lag

There are now so many reviews of the use of melatonin in jet lag, its dependence on timing and concomitant light exposure that it is pointless to write another here, see for example (98). In summary, successful studies used timed melatonin correctly, unsuccessful studies [e.g., (126)] did not. The latter in particular used a cohort who had flown from Norway to New York, stayed 4 days and then were studied on the flight back to Norway. One can predict that their study population was unadapted to New York time, phase shifted from Norwegian time, internally and externally desynchronized, and individually different since individual response to abrupt change of time cues is variable. Their lack of useful effect is not surprising since this situation was not taken into account.

In 2006 a Cochrane Database review concluded that melatonin was useful for jet lag (127). It is updated regularly. Timed melatonin treatment is recommended for jetlag by the American Academy of Sleep Medicine (128). Advice on how to time melatonin and light exposure can be found in reference (98) and elsewhere.

### Sleep in the Elderly

Sleep problems in the elderly may be due to many factors one of which may be disturbed circadian rhythms. Prolonged release melatonin “circadin” is registered for use in sleep disorder of the over 55 s. From the European Medicines Agency Website: “Circadin was more effective than placebo at improving quality of sleep and the patients' ability to function normally on the following day. When the results of all three studies were looked at together, 32% of the patients taking Circadin (86 out of 265) reported a significant improvement in symptoms after 3 weeks, compared with 19% of those taking placebo (51 out of 272).” The CHMP decided that, although Circadin has only been shown to have a small effect in a relatively small number of patients, its benefits are greater than its risks,” https://www.ema.europa.eu/en/medicines/human/EPAR/circadin.

Use of melatonin in the very elderly, particularly suffering from dementia, has been advocated but proved to have adverse effects in some studies (129).

### Sleep in Children

There has been considerable interest in the pediatric use of melatonin for sleep disorder, initially in children with neurodevelopmental disorders, in spite of the possible adverse effects on reproductive function. A large multi-center RCT has been conducted in the UK- the MENDS study (130). Escalating doses from 0.5 to 12 mg 45 min before bedtime were used. The primary outcome was sleep duration by diaries, and objective measures (actigraphy) were also used. They were able to show (130, 131) 23 min longer sleep and shortened sleep latency by 45 min. Evidently this success has inspired further treatment. Pediatric use of melatonin for sleep problems has covered autism, ADHD and intellectual disability (ID) (132) and now has expanded hugely to more general use. According to a serious UK newspaper- The Guardian- there are safety concerns: Despite the fact it is not licensed for use by any other age group, (other than over 55 s) 117,085 people under 18 were given melatonin “off label”—the term used for when a drug is given for an unapproved indication or in an unapproved age group—to aid sleep in the 2017–18 financial year. (https://www.theguardian.com/society/2018/nov/02/rise-in-melatonin-use-to-help-children-sleep-leads-to-safety-warning).

### Non 24 h Sleep Wake Cycle (Irregular Sleep Wake Cycle, Free-Running Sleep-Wake or Non-24)

This condition is the least numerous of the CRSDs but the most interesting. It is the expression of an individual circadian clock periodicity. Each person has their own periodicity usually slightly longer than 24 h (hence the tendency to delay over the weekend) which manifests itself in the absence of strong time cues, primarily the light dark cycle. Many blind people with no conscious or unconscious light perception cannot properly synchronize to the 24 h day (133–136). In expressing their own periodicity they drift away from normal clock time such that intermittently they will be in “night” phase during the day (e.g., secretion of melatonin, low alertness and performance) and day phase during the night (low melatonin, poor sleep). It has been described as a lifetime's intermittent jet lag.

**Figure 4:**
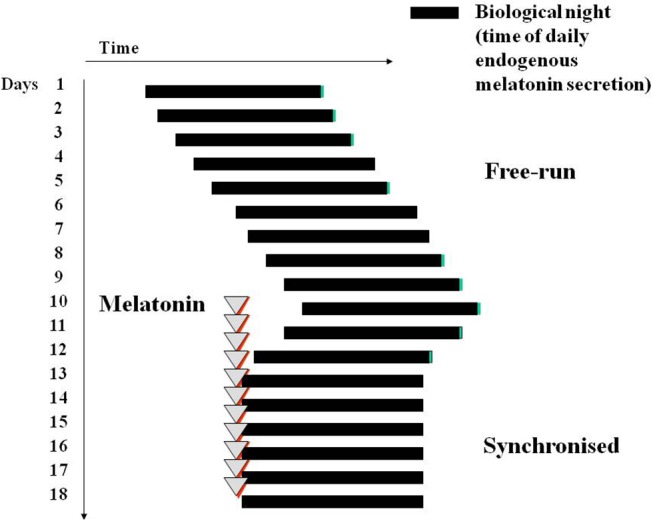
Diagram of melatonin-induced entrainment by phase advance of a free-running sleep wake cycle and circadian phase, for example in a blind subject with no conscious or unconscious light perception. Treatment is best initiated in a period of good sleep prior to desired sleep time in the “biological dusk” before onset of melatonin secretion.

Melatonin (usually 0.5–5 mg daily, lower doses than 3 mg are often better) is able to synchronize this non-24 h sleep wake cycle to 24 h in the vast majority of patients (76, 137) (Figure 4). Until a registered melatonin preparation became available in the UK (circadin) (138), patients had to obtain melatonin on a named patient basis. It is prescribed by Moorfields Eye Hospital (premier eye hospital in the UK). Unfortunately the completely blind do not often appear before a specialist, the condition is not correctly diagnosed, and is often treated with straight hypnotics (which do not work). A survey in New Zealand reported very little use of melatonin to correct this cyclic sleep disorder (139).

In 1986 a blind man rang me up, said he had non 24 h sleep wake disorder, and could he have some melatonin? He had seen my jet lag studies and worked it out for himself. After a very successful double blind placebo controlled cross over study [5 mg melatonin, (134)] he took this dose, prescribed on a named patient basis by his GP, for the rest of his life. He died 8 years ago, of prostate cancer at 83 years old, after 24 years use, refusing to lower the dose. We checked his biochemistry and hematology after 10 years treatment and all was normal for age. Since then quite a number of similar studies have found the same synchronizing effect on sleep, and from the year 2000 synchronization of the underlying circadian pacemaker was shown in most, but not all patients. The tendency is to start with very low doses and if necessary increase (or in one publication decrease) the dose until it is effective. We recommend starting treatment about an hour before bedtime when in a 'good' sleep phase. Even without full circadian entrainment sleep can be improved.

### Non-specific Insomnia

As a treatment for non-specified insomnia melatonin also appears to be quite useful. Some 3,000,000 Americans used melatonin last year according to the following website (https://nccih.nih.gov/research/statistics/NHIS/2012/natural-products/melatonin), presumably for jet lag or for “poor sleep.” In the case of non-specific “poor sleep” this is likely related in many cases to the fact that our circadian rhythms are frequently not in optimal phase in a “urban normal environment” (140) with insufficient time cues or zeitgebers to maintain optimum circadian phase. In these circumstances most people will delay the circadian system, particularly over the weekend if there is no requirement to get up in the morning. In this way the social need for sleep is in advance of the circadian optimum time and melatonin secretion in particular, and sleep suffers. The discrepancy has been referred to as “social” jet lag (141). Popping a melatonin pill in the evening has a good chance of advancing circadian phase to a more appropriate time and thus better sleep.

### Melatonin and Cancer

Animal experiments have shown clearly the increased risk of cancer with abrupt phase shifts (97, 142, 143). In 2006 the World Health Organization in Lyon, France held a week-long meeting in which, on the basis largely of animal experiments, decided that shift work was a probable carcinogen (97, 144). A large proportion of the population of developed countries (15–20%) works shifts and thus this is of major health interest.

An association of the pineal gland with anti-cancer activity has a very long history (145). Early work suggested that the gland contained oncostatic activity initially not specified as melatonin. Important evidence of the association included for example that pinealectomy of rats led to much shorter survival times from DMBA-induced cancer and secondly that exogenous melatonin could substantially increase survival times (146). In the 1970s melatonin treatment (very large doses 80–300 mg pd) to young women was proposed for avoidance (prophylactic) of breast cancer (48, 53), and development proceeded to clinical trials in combination with a progestagen. These trials were not successful. But the subject continued of interest when light at night, thought to suppress melatonin (at least partially) (147–149), was invoked as the reason for an excess of breast cancer in nurses, working rotating long term night shifts (104, 144, 150), and subsequently other shift workers.

There is good reason to consider melatonin firstly as a prophylactic, in the case of suppression by light during night work, secondly to hasten adaptation of the circadian system to abrupt phase shift when this is desirable. Thirdly it has been very extensively researched with regard to its anti-cancer activity in breast cancer and other neoplasms (151, 152). Some of the most convincing data linking physiological levels of melatonin with anti-cancer growth concerns human breast cancer-mouse xenograft studies. In a series of experiments Blask et al. could show the protective effects of exogenous and endogenous melatonin and the deleterious effects of extra light (153–156). The xenograft approach is being applied to other cancers.

Most epidemiology agrees that there is an increased risk of developing various cancers as a function of long term night shift work (97, 150). Melatonin has been used as adjuvant therapy in various cancers for nearly 20 years notably by Lissoni et al. in very advanced cancer [e.g., (157)] with positive effects but not usually significant results. With all the suggestive background, several clinical trials in different cancers using melatonin, usually as an adjunct to conventional treatment, have now been conducted (33)—but not enough. The results are quite positive in several domains- survival time, progression of the disease, reduced toxicity of treatment and in general well-being. An important question is to what extent the effects are due to rhythm optimization and/or improved sleep?

## Metabolism

A substantial early clinical literature exists concerning diurnal and ultradian rhythms in metabolic function [e.g., (158)]. With the application of constant routine technology it became possible to identify endogenously generated (i.e., circadian) rhythms from those derived from the external environment, meal times etc. (159–161). This has now been extended to metabolomics. For example simultaneous evaluation of many metabolites in constant routine has shown that of 132 circulating metabolites nearly half showed a 24 h rhythmicity (162). Following sleep deprivation it was clear that many metabolites desynchronized amongst themselves (163, 164). With sequencing of the human genome, this approach has now devolved to the level of genes (67). Melatonin has been invoked as a supplementary treatment for avoidance or reversal of metabolic syndrome but without substantial evidence of efficacy [e.g., (165)].

### The Entero-Insular Axis and Diabetes

The importance of rhythms to the entero-insular axis was also evident early on, with variations in glucose tolerance and insulin sensitivity (166). The subject has been very recently reviewed (161). The circadian, SCN-driven nature of these rhythms is now well established alongside the “masking” effects of mealtimes, meal content and other external inputs (167, 168). Triacylglycerol (TAG) has a particularly marked circadian rhythm in constant routine (167). During both simulated and real shift work, standard meals taken at inappropriate times at night—biological night when melatonin secretion is high-lead to evidence of insulin resistance/glucose intolerance and higher TAG, both risk factors for heart disease (167, 169). This is therefore one possible mechanism underlying the epidemiological data showing higher risk of these major diseases.

Circadian re-adaptation in real shift workers resolves some metabolic risk factors (169) (and see Gibbs M, Hampton SH, Morgan L, Arendt J. Effect of shift schedule on offshore shift workers' circadian rhythms and health, 2004. http://www.hse.gov.uk/research/rrhtm/rr318.htm). So there is good reason to use the chronobiotic properties of melatonin (and timed light exposure) to manipulate circadian phase. It remains to be determined to what extent central and peripheral oscillators remain in synchrony/coupled in these circumstances.

Melatonin clearly influences glucose concentrations- pinealectomy leads to increased glucose in nocturnal rats (170). In MT1 and MT2 receptor knockout mice the SCN-driven glucose rhythm is abolished independently of peripheral oscillators in muscle, adipose tissue and liver (171). In humans in one study, the decrease in glucose tolerance from morning to evening was mostly influenced by the endogenous circadian system compared to the sleep-wake cycle. However, in apparent contrast to pinealectomy effects in animals, melatonin administered during day time just prior to a glucose tolerance test in healthy adults clearly impaired glucose tolerance both in the morning and the evening (172, 173), an effect that was dependent on a common gain-of-function variant of the melatonin receptor gene MTNR1B151 (see below). Melatonin may also acutely decrease insulin secretion in cultured human islets (174). Thus, some controversy exists in the literature especially when comparing results in nocturnal rodents with diurnal humans with both beneficial and detrimental effects of melatonin reported. It is intriguing to note that the rare condition 'familial insulin resistance' or Rabden-Mendenhall syndrome is associated with pineal hyperplasia (175, 176).

In view of pre-existing associations of the pineal and melatonin with metabolic function the discovery of related MT1 and MT2 receptor variants aroused enormous interest. A common variant in MTNR1B—*MTNR1B* rs10830963 is associated with increased risk of type 2 diabetes, increased fasting plasma glucose levels and impaired early insulin secretion (177, 178). Moreover, late dinner, associated with elevated melatonin concentrations (as in night shift workers, above), impaired glucose tolerance in “gain of function” *MTNR1B* risk allele carriers but not in non-carriers. These data suggest that circulating melatonin is related to the development of Type 2 Diabetes, in a deleterious sense. Of course sleep restriction is also associated with impaired glucose tolerance, increased risk of metabolic syndrome and/or diabetes (179, 180). So that the usefulness of melatonin to address sleep problems may well increase risk of metabolic abnormalities. Some controversies have arisen and have been reviewed (181). The question is not solved.

## Cardiovascular System

Rhythmicity is a cardinal feature of the cardiovascular system, with demonstrable involvement of the SCN (182). Considerable attention has been directed at research into the disorders of rhythmic events and the timing of pharmacological interventions e.g., for elevated blood pressure (183). Timing of treatment clinically with anti-hypertensive drugs is accepted and current practice (184). Does melatonin influence the cardiovascular system? A recent review gives a positive report (185) with regard to several cardiovascular effects. In a controlled experiment melatonin was able to shift heart rate variability in company with the major circadian rhythms of cortisol, core body temperature and TSH (71). Evidently this corresponds to an effect on the central circadian clock.

There is certainly some good evidence that melatonin can lower blood pressure at night in patients with essential hypertension and/or metabolic syndrome (186, 187). Possibly the accompanying increased day night amplitude of systolic and diastolic rhythms was equally important and indicative of strengthened function of the SCN. The mechanism involved is not clear. The improved sleep reported in the subjects may well have contributed to the result.

Melatonin has probably had more exposure as a potential cardiovascular protective agent, with respect especially to myocardial ischemia/reperfusion injury. Numerous animal experiments suggest beneficial effects in a meta-analysis, with anti-oxidant effects, free-radical scavenging, anti-apoptosis and/or involvement of MT1 receptor suggested as mechanisms (188). However, a later meta-analysis and experimentation using melatonin in a combination with minocycline and magnesium sulfate did not show efficacy (189). Several clinical trials appear to be ongoing.

## Use of Melatonin as a Circadian “Marker” Rhythm, Providing Information on the Phase and Timing of the Circadian System for Basic Research, Timed Treatments

**Figure 5:**
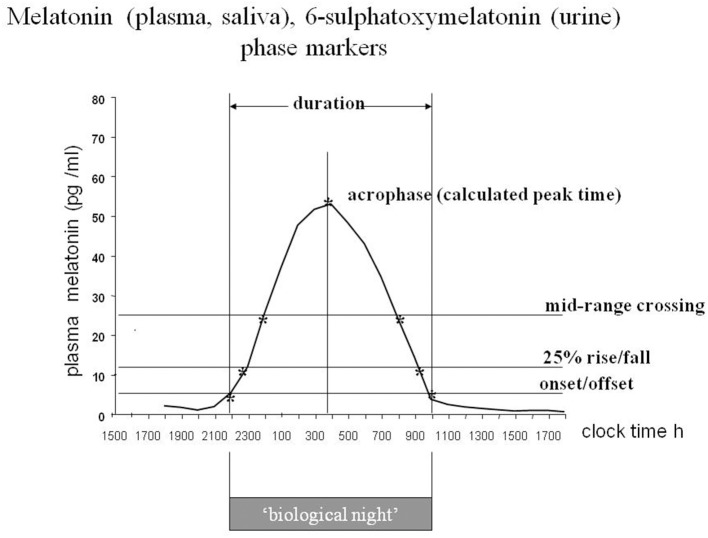
The melatonin rhythm as a marker of circadian status. Diagram of a stylized plasma or saliva melatonin or urinary 6-sulphatoxymelatonin rhythm with the characteristics that have been used to define circadian status. Each body fluid has advantages and disadvantages from a practical point of view. Plasma is the most precise, with short interval sampling, saliva and aMT6s are the most useful for field studies. For long term monitoring of circadian status urinary aMT6s is well-tolerated. From Arendt (194), by permission.

The rhythmic production of melatonin, normally high during the dark phase in all species studied to date, is linked directly via neural connections to the activity of the central circadian clock or pacemaker in the SCN (9). It was possible to show that the rate limiting synthetic enzyme pineal AA-NAT activity is closely related to the plasma melatonin profile in rats (190), and that the plasma profile is closely related to that of saliva in humans (191). Moreover, the urinary excretion of 6-sulphatoxymelatonin (aMT6s) the major metabolite in rats and humans reflects faithfully the profile of plasma melatonin in humans (192, 193). Thus, the measurable melatonin/aMT6s profiles in plasma, saliva or urine provide a ‘window’ on the clock. The melatonin rhythm has been extensively used to investigate the characteristics of human circadian rhythms. It is considered to be the best circadian marker rhythm, at least for the moment (Figure 5).

The characteristics of melatonin secretion in normal healthy volunteers have been studied for many years with increasing technological sophistication. They have been reviewed previously on numerous occasions. Similarly numerous publications describe abnormalities in melatonin secretion related to pathology. However, what is hardly ever considered is the general circadian status of patients studied. For example if a state of desynchrony exists, then an amplitude reduction in centrally driven and possibly peripheral circadian rhythms is likely (Figure 1) and low melatonin is not a specific symptom but a reflection of rhythm status. Another consideration is whether low (or high) melatonin amplitude is a cause or a consequence of the pathological state.

A change in timing of the rhythm is easier to interpret, not least because there is normally such a vast difference in amplitude between individuals (195). This is probably the feature that has been most exploited clinically- but mostly for research purposes. The complete profile with sampling at hourly intervals or less provides the most information, but the timing of the onset of secretion in the evening in dim light, known as the DLMO (the dim light melatonin onset), is convenient and has been widely used to assess circadian status (196). First it should be noted that a large change in amplitude can look like a change in DLMO, depending on how the calculations are performed. The DLMOFF (dim light melatonin offset) in the morning is also useful as a circadian marker as is the “Synoff”—the time when production ceases (197). Urinary aMT6s provides less resolution, but even with 4 h day time/waketime samples and 8 h/oversleep the calculated acrophase is within 30 min of that derived from hourly sampling (193).

Each of the 3 matrices—plasma, saliva and urine, has advantages and disadvantages. Plasma is ideal and can be done overnight during sleep but requires catheterization and volume of blood loss is important. Saliva is practical but unless the subject is woken frequently for overnight sampling, the onset can easily be missed. Sequential urine samples have lower resolution but can readily be collected and measured by subjects in field studies, include the whole profile (much preferable to an early morning urine) and carried out long term.

For example workers on North Sea oil rigs collected, measured, aliquoted and froze urine continuously for 2–3 weeks whilst on the platform (198, 199). These samples provided a continuous record of circadian adaptation to night shift, or not depending on schedule. Similarly many blind subjects (135) and the crew of an Antarctic ship (200) have collected urine for 48 h at weekly intervals for 6 weeks or more. This approach provides an evolving picture of circadian status. It was particularly important in our work to judge the timing of melatonin treatment to entrain free running rhythms in blind subjects (76) and to find out to what extent particular shift schedules onshore, offshore and in Antarctica lead to desynchrony with associated sleep and metabolic problems (63). Melatonin profiling has been extensively used in research to provide a way of normalizing experimental subjects with diverse angles of entrainment relative to the sleep wake cycle, for comparative purposes.

## Why Measure Melatonin?

In what clinical circumstances is there a need to know circadian status through melatonin measurement? Principally this is to identify desynchrony, delayed, advanced or free-running circadian status. Importantly it enables correct timing of treatment with melatonin and/or light or alternative zeitgebers as chronotherapies for disrupted rhythms, according to the appropriate PRC. Numerous drugs have large diurnal changes in pharmacokinetics which may or may not be circadian in nature (184). The important question of timing of drug treatment has become more high profile in the clinic with the individualization of treatment regimes especially for cancer and the melatonin rhythm might well provide an individual circadian marker for timing specification (201).

## The Umbrella Review

There are now so many reviews (systematic and narrative) and meta-analyses of the effects of melatonin on human and animal pathology that Posadzki et al. (33) have conducted what they refer to as “An umbrella review” or a “review of reviews” to pinpoint areas where a consensus may exist (Box 1). They identified 195 eligible articles according to their quality criteria, providing a valuable resource for evaluation of the evidence. Listed below and highly simplified are those effects and associations of melatonin in humans for which these authors found significant random effects in quantitative synthesis of eligible meta-analyses. The authors note that there is some overlap between the published meta-analyses which they identify. Furthermore, some of the analysis includes data from prolonged release melatonin circadin (two reviews) and melatonin agonists ramelteon (four reviews), agomelatine (two reviews), and tasimelteon (one review).

Box 1 **Therapeutic effects and associations of melatonin, simplified, and condensed from Pozadski et al. (33).**

In brackets: number of significant random effects/total number of analyses synthesized/total number of participants.

Risk of breast cancer up, related to low melatonin (3/3/3001)

Depression, response to treatment (1), remission (1), same study (2/6/1871)

Pre-operative anxiety down (1/2/761)

Post-operative anxiety down (1/1/73)

Post-operative pain down (1/1/524)

Prevention of agitation (1/1/170)

Safety high (1/1/2912)

Sleep latency down (3/4/6452)

Sleep quality up (2/2/5830), one study as for latency

Shown in a separate category are the significant random effects meta-analyses with insufficient data for quantitative synthesis:

In brackets: number of significant analyses/total number of subjects.

Breast cancer, risk of death at 1 year down (13 studies but no information on total number of subjects)

Nocturnal hypertension, systolic and diastolic, down (3/72)

Sleep latency down (5/2234)

Sleep duration up (4/2417), largest study significant for latency as above but non-significant for duration)

Melatonin onset (DLMO) (6/238)

Core temperature down (16/193)

A meta-analysis of the protective effects of melatonin in ischemic stroke in rodents is included with 432 animals and a highly significant large effect size.

The conclusion from this tour de force is that the data supports the notion that endogenous and exogenous melatonin has benefits for health- a conclusion with which most people would agree. However, given the vast number of potential therapeutic uses for this hormone (as opposed to the accepted uses in sleep disorder) there are very few sufficiently large, randomized, multi-center, placebo-controlled, double blind trials in specific applications. Hopefully more are on the way.

## Concluding Remarks

It seems that there are two major schools of research into the clinical therapeutic effects of melatonin. Firstly its association with biological rhythms from cells to organisms, and particularly with the timing and quality of sleep. It acts via well-characterized membrane receptors, which will be considered in depth by others in this volume.

Secondly in the last 25 years the expanding field of protective effects, often considered not to require receptor signaling, has come into being. From a philosophical point of view it would be very satisfying to reconcile these two approaches. It is probably true to say that “everything is rhythmic unless proved otherwise.” If so, melatonin as a rhythm optimizer (synchronization, re-synchronization, entrainment, re-entrainment, coupling, phase and amplitude adjustment, phase and amplitude maintenance, periodicity), could well be invoked to explain many, even most therapeutic protective effects. Or at least a part of these effects.

It has been referred to as “circadian glue” but its influence on rhythms extends to other periodicities. Moreover, its “beneficial” effects on sleep may lead to a multitude of downstream events of therapeutic value. For example reducing the risk of insulin resistance, metabolic syndrome, obesity, diabetes, all of which have been associated with poor and/or insufficient sleep (see text). The association of melatonin with risk of cancer and therapeutic intervention therein, is strongly related to disordered rhythms.

Melatonin is good for human health, particularly via its ability to optimize sleep timing and often duration and quality with a multitude of downstream benefits. It can counter the debilitating effects of modern lifestyles: insufficient circadian time cues especially natural bright light, and exposure to artificial light at unsuitable times—the 24 h society. It is questionable whether or not long term use has deleterious effects and this is particularly important in pediatrics.

Circadian and other rhythm status, and reproductive function, during treatment with very large doses of melatonin needs investigation.

Finally, from personal anecdotal evidence, having taken melatonin in 2–5 mg oral fast release formulation on and off, mostly on, since 1981 after a mastectomy, I know that it does not prevent some of the pathologies associated with old age—osteoarthritis, Type II diabetes, spinal stenosis, uterine cancer. But I am still here!

## Data Availability

All relevant data analyzed are included in this manuscript.

## Author Contributions

The author confirms being the sole contributor of this work and has approved it for publication.

### Conflict of Interest Statement

JA is director of two companies Stockgrand Ltd and Surrey Assays Ltd which are concerned with measuring melatonin, 6-sulphatoxymelatonin, and other hormones. These companies had no influence on the writing of this text.

## References

[1] Arendt J. Melatonin and the Mammalian Pineal Gland. London: Chapman Hall (1995).

[2] Jockers R Delagrange P Dubocovich ML Markus RP Renault N Tosini G. Update on melatonin receptors: IUPHAR Review 20. Br J Pharmacol. (2016) 173:2702–25. 10.1111/bph.13536 27314810 PMC4995287

[3] Liu J Clough SJ Hutchinson AJ Adamah-Biassi EB Popovska-Gorevski M Dubocovich ML. MT1 and MT2 melatonin receptors: a therapeutic perspective. Annu Rev Pharmacol Toxicol. (2016) 56:361–83. 10.1146/annurev-pharmtox-010814-124742 26514204 PMC5091650

[4] Olcese J Lozier S Paradise C. Melatonin and the circadian timing of human parturition. Reprod Sci. (2013) 20:168–74. 10.1177/1933719112442244 22556015

[5] Olcese J Beesley S. Clinical significance of melatonin receptors in the human myometrium. Fertil Steril. (2014) 102:329–35. 10.1016/j.fertnstert.2014.06.020 25015556

[6] Rahman SA Bibbo C Olcese J Czeisler CA Robinson JN Klerman EB. Relationship between endogenous melatonin concentrations and uterine contractions in late third trimester of human pregnancy. J Pineal Res. (2019) 66:e12566. 10.1111/jpi.12566 30739346 PMC6453747

[7] Wehr TA Moul DE Barbato G Giesen HA Seidel JA Barker C. Conservation of photoperiod-responsive mechanisms in humans. Am J Physiol. (1993) 265(4 Pt 2):R846–57. 10.1152/ajpregu.1993.265.4.R846 8238456

[8] Sáenz de Miera C Sage-Ciocca D Simonneaux V Pévet P Monecke S. Melatonin-independent photoperiodic entrainment of the circannual TSH rhythm in the pars tuberalis of the European hamster. J. Biol. Rhythms. (2018) 33:302–17. 10.1177/0748730418766601 29618281

[9] Klein DC. Photoneural regulation of the mammalian pineal gland. Ciba Found Symp. (1985) 117:38–56. 10.1002/9780470720981.ch4 3015512

[10] Klein DC. Arylalkylamine N-acetyltransferase: the timezyme. J Biol Chem. (2007) 282:4233–7. 10.1074/jbc.R600036200 17164235

[11] Lewy AJ Wehr TA Goodwin FK Newsome DA Markey SP. Light suppresses melatonin secretion in humans. Science. (1980) 210:1267–9. 10.1126/science.7434030 7434030

[12] Bojkowski CJ Aldhous ME English J Franey C Poulton AL Skene DJ. Suppression of nocturnal plasma melatonin and 6-sulphatoxymelatonin by bright and dim light in man. Horm Metab Res. (1987) 19:437–40. 10.1055/s-2007-1011846 3692439

[13] Zeitzer JM Dijk DJ Kronauer R Brown E Czeisler C. Sensitivity of the human circadian pacemaker to nocturnal light: melatonin phase resetting and suppression. J Physiol. (2000) 526(Pt 3):695–702. 10.1111/j.1469-7793.2000.00695.x 10922269 PMC2270041

[14] Berson DM Dunn FA Takao M. Phototransduction by retinal ganglion cells that set the circadian clock. Science. (2002) 295:1070–3. 10.1126/science.1067262 11834835

[15] Lucas RJ Peirson SN Berson DM Brown TM Cooper HM Czeisler CA. Measuring and using light in the melanopsin age. Trends Neurosci. (2014) 37:1–9. 10.1016/j.tins.2013.10.004 24287308 PMC4699304

[16] Hirata F Hayaishi O Tokuyama T Seno S. *In vitro* and *in vivo* formation of two new metabolites of melatonin. J Biol Chem. (1974) 249:1311–3. 4814344

[17] Ma X Idle JR Krausz KW Tan DX Ceraulo L Gonzalez FJ. Urinary metabolites and antioxidant products of exogenous melatonin in the mouse. J Pineal Res. (2006) 40:343–9. 10.1111/j.1600-079X.2006.00321.x 16635022 PMC1448215

[18] Lane EA Moss HB. Pharmacokinetics of melatonin in man: first pass hepatic metabolism. J Clin Endocrinol Metab. (1985) 61:1214–6. 10.1210/jcem-61-6-1214 4055987

[19] Rajaratnam SM Dijk DJ Middleton B Stone BM Arendt J. Melatonin phase-shifts human circadian rhythms with no evidence of changes in the duration of endogenous melatonin secretion or the 24-hour production of reproductive hormones. J Clin Endocrinol Metab. (2003) 88:4303–9. 10.1210/jc.2003-030460 12970302

[20] Zetner D Andersen LP Rosenberg J. Pharmacokinetics of alternative administration routes of melatonin: a systematic review. Drug Res. (2016) 66:169–73. 10.1055/s-0035-1565083 26514093

[21] Sugden D. Psychopharmacological effects of melatonin in mouse and rat. J Pharmacol Exp Ther. (1983) 227:587–91. 6655558

[22] Guardiola-Lemaître B. Toxicology of melatonin. J Biol Rhythms. (1997) 12:697–706. 10.1177/074873049701200627 9406047

[23] Arendt J Forbes M Brown W Marston A Effect of pinealectomy on immunoassayable melatonin in sheep. J Endocrinol. (1980) 85:1.6156224

[24] Lewy AJ Tetsuo M Markey SP Goodwin FK Kopin IJ. Pinealectomy abolishes plasma melatonin in the rat. J Clin Endocrinol Metab. (1980) 50:204–5. 10.1210/jcem-50-1-204 7350183

[25] Bittman EL Karsch FJ Hopkins JW. Role of the pineal gland in ovine photoperiodism: regulation of seasonal breeding and negative feedback effects of estradiol upon luteinizing hormone secretion. Endocrinology. (1983) 113:329–36. 10.1210/endo-113-1-329 6861705

[26] Djeridane Y Vivien-Roels B Simonneaux V Miguez JM Pévet P. Evidence for melatonin synthesis in rodent Harderian gland: a dynamic in vitro study. J Pineal Res. (1998) 25:54–64. 10.1111/j.1600-079X.1998.tb00386.x 9694405

[27] Konturek SJ Konturek PC Brzozowski T Bubenik GA. Role of melatonin in upper gastrointestinal tract. J Physiol Pharmacol. (2007) 58(Suppl. 6):23–52. 18212399

[28] Venegas C García JA Escames G Ortiz F López A Doerrier C. Extrapineal melatonin: analysis of its subcellular distribution and daily fluctuations. J Pineal Res. (2012) 52:217–27. 10.1111/j.1600-079X.2011.00931.x 21884551

[29] Acuña-Castroviejo D Escames G Venegas C Díaz-Casado ME Lima-Cabello E López LC. Extrapineal melatonin: sources, regulation, and potential functions. Cell Mol Life Sci. (2014) 71:2997–3025. 10.1007/s00018-014-1579-2 24554058 PMC11113552

[30] Lincoln GA Short RV. Seasonal breeding: nature's contraceptive. Recent Prog Horm Res. (1980) 36:1–52. 10.1016/B978-0-12-571136-4.50007-3 6774387

[31] Fukuhara C Liu C Ivanova TN Chan GC Storm DR Iuvone PM. Gating of the cAMP signaling cascade and melatonin synthesis by the circadian clock in mammalian retina. J Neurosci. (2004) 24:1803–11. 10.1523/JNEUROSCI.4988-03.2004 14985420 PMC6730387

[32] Iuvone PM Boatright JH Tosini G Ye K. N-acetylserotonin: circadian activation of the BDNF receptor and neuroprotection in the retina and brain. Adv Exp Med Biol. (2014) 801:765–71. 10.1007/978-1-4614-3209-8_96 24664769 PMC4069859

[33] Posadzki PP Bajpai R Kyaw BM Roberts NJ Brzezinski A Christopoulos GI. Melatonin and health: an umbrella review of health outcomes and biological mechanisms of action. BMC Med. (2018) 16:18. 10.1186/s12916-017-1000-8 29397794 PMC5798185

[34] Suofu Y Li W Jean-Alphonse FG Jia J Khattar NK Li J. Dual role of mitochondria in producing melatonin and driving GPCR signaling to block cytochrome c release. Proc Natl Acad Sci USA. (2017) 114:E7997–8006. 10.1073/pnas.1705768114 28874589 PMC5617277

[35] Paulose JK Cassone VM. The melatonin-sensitive circadian clock of the enteric bacterium *Enterobacter aerogenes*. Gut Microbes. (2016) 7:424–7. 10.1080/19490976.2016.1208892 27387841 PMC5154366

[36] Dubbels R Reiter RJ Klenke E Goebel A Schnakenberg E Ehlers C. Melatonin in edible plants identified by radioimmunoassay and by high performance liquid chromatography-mass spectrometry. J Pineal Res. (1995) 18:28–31. 10.1111/j.1600-079X.1995.tb00136.x 7776176

[37] Gerdin MJ Masana MI Ren D Miller RJ Dubocovich ML. Short-term exposure to melatonin differentially affects the functional sensitivity and trafficking of the hMT1 and hMT2 melatonin receptors. J Pharmacol Exp Ther. (2003) 304:931–9. 10.1124/jpet.102.044990 12604667

[38] Gerdin MJ Masana MI Rivera-Bermúdez MA Hudson RL Earnest DJ Gillette MU. Melatonin desensitizes endogenous MT2 melatonin receptors in the rat suprachiasmatic nucleus: relevance for defining the periods of sensitivity of the mammalian circadian clock to melatonin. FASEB J. (2004) 18:1646–56. 10.1096/fj.03-1339com 15522910

[39] Goldman BD. Mammalian photoperiodic system: formal properties and neuroendocrine mechanisms of photoperiodic time measurement. J Biol Rhythms. (2001) 16:283–301. 10.1177/074873001129001980 11506375

[40] Lincoln GA. Neuroendocrine regulation of seasonal gonadotrophin and prolactin rhythms: lessons from the soay ram model. Reprod Suppl. (2002) 59:131–47. 12698978

[41] Dardente H. Melatonin-dependent timing of seasonal reproduction by the pars tuberalis: pivotal roles for long daylengths and thyroid hormones. J Neuroendocrinol. (2012) 24:249–66. 10.1111/j.1365-2826.2011.02250.x 22070540

[42] Lincoln G Loudon A. Looking inside the seasonal clock. J Neuroendocrinol. (2015) 27:76–7. 10.1111/jne.12238 25529073

[43] Chemineau P Malpaux B. [Melatonin and reproduction in domestic farm animals]. Therapie. (1998) 53:445–52. 9921036

[44] Rosenthal NE Sack DA Gillin JC Lewy AJ Goodwin FK Davenport Y. Seasonal affective disorder. a description of the syndrome and preliminary findings with light therapy. Arch Gen Psychiatry. (1984) 41:72–80. 10.1001/archpsyc.1984.01790120076010 6581756

[45] Wirz-Justice A Terman M. Chronotherapeutics (light and wake therapy) as a class of interventions for affective disorders. Handb Clin Neurol. (2012) 106:697–713. 10.1016/B978-0-444-52002-9.00042-5 22608653

[46] Martin JE McKellar S Klein DC. Melatonin inhibition of the in vivo pituitary response to luteinizing hormone-releasing hormone in the neonatal rat. Neuroendocrinology. (1980) 31:13–7. 10.1159/000123044 6993981

[47] Puig-Domingo M Webb SM Serrano J Peinado MA Corcoy R Ruscalleda J. Brief report: melatonin-related hypogonadotropic hypogonadism. N Engl J Med. (1992) 327:1356–9. 10.1056/NEJM199211053271905 1406837

[48] Voordouw BC Euser R Verdonk RE Alberda BT de Jong FH Drogendijk AC. Melatonin and melatonin-progestin combinations alter pituitary-ovarian function in women and can inhibit ovulation. J Clin Endocrinol Metab. (1992) 74:108–17. 10.1210/jcem.74.1.1727807 1727807

[49] Luboshitsky R Lavie P. Early morning melatonin levels in hypogonadal men. J Clin Endocrinol Metab. (1996) 81:4181–2. 10.1210/jc.81.11.4181 8923887

[50] Yellon SM Foster DL. Melatonin rhythms time photoperiod-induced puberty in the female lamb. Endocrinology. (1986) 119:44–9. 10.1210/endo-119-1-44 3720672

[51] Vanecek J Klein DC. Melatonin inhibits gonadotropin-releasing hormone-induced elevation of intracellular Ca2+ in neonatal rat pituitary cells. Endocrinology. (1992) 130:701–7. 10.1210/en.130.2.701 1733718

[52] Arendt J Labib MH Bojkowski C Hanson S Marks V. Rapid decrease in melatonin production during successful treatment of delayed puberty. Lancet. (1989) 1:1326. 10.1016/S0140-6736(89)92716-5 2566850

[53] Cohen M Small RA Brzezinski A. Hypotheses: melatonin/steroid combination contraceptives will prevent breast cancer. Breast Cancer Res Treat. (1995) 33:257–64. 10.1007/BF00665950 7749153

[54] Warren WS Cassone VM. The pineal gland: photoreception and coupling of behavioral, metabolic, and cardiovascular circadian outputs. J Biol Rhythms. (1995) 10:64–79. 10.1177/074873049501000106 7632982

[55] Fisher SP Sugden D Endogenous melatonin is not obligatory for the regulation of the rat sleep-wake cycle. Sleep. (2010) 33:833–40. 10.1093/sleep/33.6.833 20550025 PMC2881717

[56] Quay WB. Precocious entrainment and associated characteristics of activity patterns following pinalectomy and reversal of photoperiod. Physiol Behav. (1970) 5:1281–90. 10.1016/0031-9384(70)90041-7 5524512

[57] Armstrong SM Redman J. Melatonin administration: effects on rodent circadian rhythms. Ciba Found Symp. (1985) 117:188–207. 3836814 10.1002/9780470720981.ch12

[58] Deacon S English J Tate J Arendt J. Atenolol facilitates light-induced phase shifts in humans. Neurosci Lett. (1998) 242:53–6. 10.1016/S0304-3940(98)00024-X 9510003

[59] Dijk DJ Duffy JF Riel E Shanahan TL Czeisler CA. Ageing and the circadian and homeostatic regulation of human sleep during forced desynchrony of rest, melatonin and temperature rhythms. J Physiol. (1999) 516(Pt 2):611–27. 10.1111/j.1469-7793.1999.0611v.x 10087357 PMC2269279

[60] Slawik H Stoffel M Riedl L Veselý Z Behr M Lehmberg J. Prospective Study on Salivary Evening Melatonin and Sleep before and after Pinealectomy in Humans. J. Biol. Rhythms. (2016) 31:82–93. 10.1177/0748730415616678 26647380

[61] Rajaratnam SM Arendt J. Health in a 24-h society. Lancet. (2001) 358:999–1005. 10.1016/S0140-6736(01)06108-6 11583769

[62] Akerstedt T. Shift work and sleep disorders. Sleep. (2005) 28:9–11. 15700712

[63] Arendt J. Shift work: coping with the biological clock. Occup Med. (2010) 60:10–20. 10.1093/occmed/kqp162 20051441

[64] Ando H Fujimura A. [Circadian clock disruption and diabetes mellitus]. Nippon Rinsho. (2013) 71:2114–8. 24437264

[65] Archer SN Laing EE Moller-Levet CS van der Veen DR Bucca G Lazar AS. Mistimed sleep disrupts circadian regulation of the human transcriptome. Proc Natl Acad Sci USA. (2014) 111:E682–91. 10.1073/pnas.1316335111 24449876 PMC3926083

[66] Broussard JL Van Cauter E. Disturbances of sleep and circadian rhythms: novel risk factors for obesity. Curr Opin Endocrinol Diabetes Obes. (2016) 23:353–9. 10.1097/MED.0000000000000276 27584008 PMC5070789

[67] Panda S. The arrival of circadian medicine. Nat Rev Endocrinol. (2019) 15:67–9. 10.1038/s41574-018-0142-x 30602736

[68] Arendt J Skene DJ. Melatonin as a chronobiotic. Sleep Med Rev. (2005) 9:25–39. 10.1016/j.smrv.2004.05.002 15649736

[69] Middleton B Rajaratnam SMW Stone B Dijk D-J Arendt J Hormonal response to a melatonin-induced shift in sleep. J Sleep Res. (2002). 11:154.

[70] Rajaratnam SM Middleton B Stone BM Arendt J Dijk DJ. Melatonin advances the circadian timing of EEG sleep and directly facilitates sleep without altering its duration in extended sleep opportunities in humans. J Physiol. (2004) 561(Pt 1):339–51. 10.1113/jphysiol.2004.073742 15459246 PMC1665336

[71] Vandewalle G Middleton B Rajaratnam SM Stone BM Thorleifsdottir B Arendt J. Robust circadian rhythm in heart rate and its variability: influence of exogenous melatonin and photoperiod. J Sleep Res. (2007) 16:148–55. 10.1111/j.1365-2869.2007.00581.x 17542944

[72] Lewy AJ Bauer VK Ahmed S Thomas KH Cutler NL Singer CM. The human phase response curve (PRC) to melatonin is about 12 hours out of phase with the PRC to light. Chronobiol Int. (1998) 15:71–83. 10.3109/07420529808998671 9493716

[73] Burgess HJ Sharkey KM Eastman CI. Bright light, dark and melatonin can promote circadian adaptation in night shift workers. Sleep Med Rev. (2002) 6:407–20. 10.1053/smrv.2001.0215 12531129

[74] Paul MA Gray GW Lieberman HR Love RJ Miller JC Trouborst M. Phase advance with separate and combined melatonin and light treatment. Psychopharmacology. (2011) 214:515–23. 10.1007/s00213-010-2059-5 21069516

[75] Deacon S Arendt J. Melatonin-induced temperature suppression and its acute phase-shifting effects correlate in a dose-dependent manner in humans. Brain Res. (1995) 688:77–85. 10.1016/0006-8993(95)96872-I 8542325

[76] Lockley SW Skene DJ James K Thapan K Wright J Arendt J. Melatonin administration can entrain the free-running circadian system of blind subjects. J Endocrinol. (2000) 164:R1–6. 10.1677/joe.0.164r001 10607943

[77] Sack RL Brandes RW Kendall AR Lewy AJ. Entrainment of free-running circadian rhythms by melatonin in blind people. N Engl J Med. (2000) 343:1070–7. 10.1056/NEJM200010123431503 11027741

[78] Middleton B Arendt J Stone BM. Complex effects of melatonin on human circadian rhythms in constant dim light. J Biol Rhythms. (1997) 12:467–77. 10.1177/074873049701200508 9376645

[79] McArthur AJ Gillette MU Prosser RA. Melatonin directly resets the rat suprachiasmatic circadian clock in vitro. Brain Res. (1991) 565:158–61. 10.1016/0006-8993(91)91748-P 1773352

[80] Reppert SM. Melatonin receptors: molecular biology of a new family of G protein-coupled receptors. J Biol Rhythms. (1997) 12:528–31. 10.1177/074873049701200606 9406026

[81] Dubocovich ML. Melatonin receptors: role on sleep and circadian rhythm regulation. Sleep Med. (2007) 8(Suppl. 3):34–42. 10.1016/j.sleep.2007.10.007 18032103

[82] Klosen P Lapmanee S Schuster C Guardiola B Hicks D Pevet P. MT1 and MT2 melatonin receptors are expressed in nonoverlapping neuronal populations. J Pineal Res. (2019). 10.1111/jpi.12575. [Epub ahead of print]. 30937953

[83] Li J Hand LE Meng QJ Loudon AS Bechtold DA. GPR50 interacts with TIP60 to modulate glucocorticoid receptor signalling. PLoS ONE. (2011) 6:e23725. 10.1371/journal.pone.0023725 21858214 PMC3157439

[84] Cuesta M Cermakian N Boivin DB. Glucocorticoids entrain molecular clock components in human peripheral cells. FASEB J. (2015) 29:1360–70. 10.1096/fj.14-265686 25500935

[85] Agez L Laurent V Pévet P Masson-Pévet M Gauer F. Melatonin affects nuclear orphan receptors mRNA in the rat suprachiasmatic nuclei. Neuroscience. (2007) 144:522–30. 10.1016/j.neuroscience.2006.09.030 17067745

[86] Hablitz LM Molzof HE Abrahamsson KE Cooper JM Prosser RA Gamble KL. GIRK channels mediate the nonphotic effects of exogenous melatonin. J Neurosci. (2015) 35:14957–65. 10.1523/JNEUROSCI.1597-15.2015 26558769 PMC4642232

[87] Johnston JD Tournier BB Andersson H Masson-Pévet M Lincoln GA Hazlerigg DG. Multiple effects of melatonin on rhythmic clock gene expression in the mammalian pars tuberalis. Endocrinology. (2006) 147:959–65. 10.1210/en.2005-1100 16269454

[88] Valenzuela FJ Torres-Farfan C Richter HG Mendez N Campino C Torrealba F. Clock gene expression in adult primate suprachiasmatic nuclei and adrenal: is the adrenal a peripheral clock responsive to melatonin? Endocrinology. (2008) 149:1454–61. 10.1210/en.2007-1518 18187542

[89] Zeman M Herichova I. Melatonin and clock genes expression in the cardiovascular system. Front Biosci. (2013) 5:743–53. 10.2741/S404 23277083

[90] Sandu C Liu T Malan A Challet E Pévet P Felder-Schmittbuhl MP. Circadian clocks in rat skin and dermal fibroblasts: differential effects of aging, temperature and melatonin. Cell Mol Life Sci. (2015) 72:2237–48. 10.1007/s00018-014-1809-7 25563487 PMC11113462

[91] Torres-Farfan C Rocco V Monsó C Valenzuela FJ Campino C Germain A. (2006). Maternal melatonin effects on clock gene expression in a nonhuman primate fetus. Endocrinology. 147:4618–26. 10.1210/en.2006-0628 16840546

[92] Jung-Hynes B Ahmad N. SIRT1 controls circadian clock circuitry and promotes cell survival: a connection with age-related neoplasms. FASEB J. (2009) 23:2803–9. 10.1096/fj.09-129148 19439501 PMC2796903

[93] Arendt J. Melatonin and the pineal gland: influence on mammalian seasonal and circadian physiology. Rev Reprod. (1998) 3:13–22. 10.1530/ror.0.0030013 9509985

[94] Illnerová H Zvolsky P Vanecek J. The circadian rhythm in plasma melatonin concentration of the urbanized man: the effect of summer and winter time. Brain Res. (1985) 328:186–9. 10.1016/0006-8993(85)91342-3 3971177

[95] Arendt J. Biological rhythms during residence in polar regions. Chronobiol Int. (2012) 29:379–94. 10.3109/07420528.2012.668997 22497433 PMC3793275

[96] de la Iglesia HO Moreno C Lowden A Louzada F Marqueze E Levandovski R. Ancestral sleep. Curr. Biol. (2016) 26:R271–2. 10.1016/j.cub.2016.01.071 27046809

[97] Straif K Baan R Grosse Y Secretan B El Ghissassi F Bouvard V. Carcinogenicity of shift-work, painting, and fire-fighting. Lancet Oncol. (2007) 8:1065–6. 10.1016/S1470-2045(07)70373-X 19271347

[98] Arendt J. Managing jet lag: some of the problems and possible new solutions. Sleep Med Rev. (2009) 13:249–56. 10.1016/j.smrv.2008.07.011 19147377

[99] Dijk D-J Duffy JF Silva EJ Shanahan TL Boivin DB Czeisler CA. Amplitude reduction and phase shifts of melatonin, cortisol and other circadian rhythms after a gradual advance of sleep and light exposure in humans. PLoS ONE. (2012) 7:e30037. 10.1371/journal.pone.0030037 22363414 PMC3281823

[100] Challet E. Circadian clocks, food intake, and metabolism. Prog Mol Biol Trans Sci. (2013) 119:105–35. 10.1016/B978-0-12-396971-2.00005-1 23899596

[101] Garaulet M Gomez-Abellan P. Chronobiology and obesity. Nutri Hospital. (2013) 28(Suppl. 5):114–20. 10.1007/978-1-4614-5082-5 24010751

[102] Ferrell JM Chiang JYL Short-term circadian disruption Impairs bile acid and lipid homeostasis in mce. Cell Mol Gastroenterol Hepatol. (2015) 1:664–77. 10.1016/j.jcmgh.2015.08.003 26645046 PMC4669895

[103] Buijs FN Leon-Mercado L Guzman-Ruiz M Guerrero-Vargas NN Romo-Nava F Buijs RM. The circadian system: a regulatory feedback network of periphery and brain. Physiology. (2016) 31:170–81. 10.1152/physiol.00037.2015 27053731

[104] Stevens RG. Light-at-night, circadian disruption and breast cancer: assessment of existing evidence. Int J Epidemiol. (2009) 38:963–70. 10.1093/ije/dyp178 19380369 PMC2734067

[105] Haus EL Smolensky MH. Shift work and cancer risk: potential mechanistic roles of circadian disruption, light at night, and sleep deprivation. Sleep Med Rev. (2013) 17:273–84. 10.1016/j.smrv.2012.08.003 23137527

[106] Schernhammer ES Schulmeister K. Melatonin and cancer risk: does light at night compromise physiologic cancer protection by lowering serum melatonin levels? Br J Cancer. (2004) 90:941–3. 10.1038/sj.bjc.6601626 14997186 PMC2409637

[107] Schernhammer ES Hankinson SE. Urinary melatonin levels and breast cancer risk. J Natl Cancer Inst. (2005) 97:1084–7. 10.1093/jnci/dji190 16030307

[108] Williams WP McLin DE Dressman MA Neubauer DN. Comparative review of approved melatonin agonists for the treatment of circadian rhythm sleep-wake disorders. Pharmacotherapy. (2016) 36:1028–41. 10.1002/phar.1822 27500861 PMC5108473

[109] Cagnacci A Elliott JA Yen SS. Melatonin: a major regulator of the circadian rhythm of core temperature in humans. J Clin Endocrinol Metab. (1992) 75:447–52. 10.1210/jc.75.2.447 1639946

[110] Cagnacci A Kräuchi K Wirz-Justice A Volpe A. Homeostatic versus circadian effects of melatonin on core body temperature in humans. J Biol Rhythms. (1997) 12:509–17. 10.1177/074873049701200604 9406024

[111] Kräuchi K Cajochen C Wirz-Justice A. A relationship between heat loss and sleepiness: effects of postural change and melatonin administration. J Appl Physiol. (1997) 83:134–9. 10.1152/jappl.1997.83.1.134 9216955

[112] Arendt J Borbely AA Franey C Wright J. The effects of chronic, small doses of melatonin given in the late afternoon on fatigue in man: a preliminary study. Neurosci Lett. (1984) 45:317–21. 10.1016/0304-3940(84)90245-3 6728321

[113] Wehr TA Aeschbach D Duncan WC. Evidence for a biological dawn and dusk in the human circadian timing system. J Physiol. (2001) 535(Pt 3):937–51. 10.1111/j.1469-7793.2001.t01-1-00937.x 11559786 PMC2278827

[114] Bjorvatn B Pallesen S. A practical approach to circadian rhythm sleep disorders. Sleep Med Rev. (2009) 13:47–60. 10.1016/j.smrv.2008.04.009 18845459

[115] Buscemi N Vandermeer B Hooton N Pandya R Tjosvold L Hartling L. Efficacy and safety of exogenous melatonin for secondary sleep disorders and sleep disorders accompanying sleep restriction: meta-analysis. Br Med J. (2006) 332:385–8. 10.1136/bmj.38731.532766.F6 16473858 PMC1370968

[116] Van Geijlswijk IM Korzilius HPLM Smits MG. The use of exogenous melatonin in delayed sleep phase disorder: a meta-analysis. Sleep. (2010) 33:1605–14. 10.1093/sleep/33.12.1605 21120122 PMC2982730

[117] Sack RL Auckley D Auger RR Carskadon MA Wright KP Vitiello MV. Circadian rhythm sleep disorders: part II, advanced sleep phase disorder, delayed sleep phase disorder, free-running disorder, and irregular sleep-wake rhythm. Am Acad Sleep Med Rev Sleep. (2007) 30:1484–501. 10.1093/sleep/30.11.1484 18041481 PMC2082099

[118] Saxvig IW Wilhelmsen-Langeland A Pallesen S Vedaa O Nordhus IH Bjorvatn B. A randomized controlled trial with bright light and melatonin for delayed sleep phase disorder: effects on subjective and objective sleep. Chronobiol Int. (2014) 31:72–86. 10.3109/07420528.2013.823200 24144243

[119] Sletten TL Magee M Murray JM Gordon CJ Lovato N Kennaway DJ. Efficacy of melatonin with behavioural sleep-wake scheduling for delayed sleep-wake phase disorder: a double-blind, randomised clinical trial. PLoS Med. (2018) 15:e1002587. 10.1371/journal.pmed.1002587 29912983 PMC6005466

[120] Murray JM Sletten TL Magee M Gordon C Lovato N Bartlett DJ. Prevalence of circadian misalignment and its association with depressive symptoms in delayed sleep phase disorder. Sleep. (2017) 40:zsw002. 10.1093/sleep/zsw002 28364473

[121] Folkard S Arendt J Clark M. Can melatonin improve shift workers' tolerance of the night shift? Some Prelim Find Chronobiol Int. (1993) 10:315–20. 10.3109/07420529309064485 8261530

[122] Bjorvatn B Stangenes K Øyane N Forberg K Lowden A Holsten F. Randomized placebo-controlled field study of the effects of bright light and melatonin in adaptation to night work. Scand J Work Environ Health. (2007) 33:204–14. 10.5271/sjweh.1129 17572830

[123] Revell VL Burgess HJ Gazda CJ Smith MR Fogg LF Eastman CI. Advancing human circadian rhythms with afternoon melatonin and morning intermittent bright light. J Clin Endocrinol Metab. (2006) 91:54–9. 10.1210/jc.2005-1009 16263827 PMC3841985

[124] Smith MR Fogg LF Eastman CI. Practical interventions to promote circadian adaptation to permanent night shift work: study 4. J Biol Rhythms. (2009) 24:161–72. 10.1177/0748730409332068 19346453

[125] Zee PC Goldstein CA. Treatment of shift work disorder and jet lag. Curr Treat Options Neurol. (2010) 12:396–411. 10.1007/s11940-010-0090-9 20842597

[126] Spitzer RL Terman M Williams JB Terman JS Malt UF Singer F. Jet lag: clinical features, validation of a new syndrome-specific scale, and lack of response to melatonin in a randomized, double-blind trial. Am J Psychiatry. (1999) 156:1392–6. 10484950 10.1176/ajp.156.9.1392

[127] Herxheimer A Petrie KJ Melatonin for preventing and treating jet lag. Cochrane Database Syst Rev. (2001). 10.1002/14651858.CD001520 11279722 PMC8958662

[128] Morgenthaler TI Lee-Chiong T Alessi C Friedman L Aurora RN Boehlecke B. Practice parameters for the clinical evaluation and treatment of circadian rhythm sleep disorders. Am Acad Sleep Med Rep Sleep. (2007) 30:1445–59. 10.1093/sleep/30.11.1445 18041479 PMC2082098

[129] Riemersma-van der Lek RF Swaab DF Twisk J Hol EM Hoogendijk WJ Van Someren EJ. Effect of bright light and melatonin on cognitive and noncognitive function in elderly residents of group care facilities: a randomized controlled trial. JAMA. (2008) 299:2642–55. 10.1001/jama.299.22.2642 18544724

[130] Appleton R Jones A Gamble C Williamson P Wiggs L Montgomery P. The use of melatonin in children with neurodevelopmental disorders and impaired sleep: a randomised, double-blind, placebo-controlled, parallel study (MENDS). Health Technol Assess. (2012) 16:1–239. 10.3310/hta16400 23098680

[131] Appleton RE Gringras P. Melatonin: helping to MEND impaired sleep. Arch Dis Child. (2013) 98:216–7. 10.1136/archdischild-2012-303606 23413423

[132] Gringras P Nir T Breddy J Frydman-Marom A Findling RL. Efficacy and safety of pediatric prolonged-release melatonin for insomnia in children with autism spectrum disorder. J Am Acad Child Adolesc Psychiatry. (2017) 56:948–57.e944. 10.1016/j.jaac.2017.09.414 29096777

[133] Lewy AJ Newsome DA. Different types of melatonin circadian secretory rhythms in some blind subjects. J Clin Endocrinol Metab. (1983) 56:1103–7. 10.1210/jcem-56-6-1103 6841552

[134] Arendt J Aldhous M Wright J. Synchronisation of a disturbed sleep-wake cycle in a blind man by melatonin treatment. Lancet. (1988) 1:772–3. 10.1016/S0140-6736(88)91586-3 2895305

[135] Skene DJ Arendt J. Circadian rhythm sleep disorders in the blind and their treatment with melatonin. Sleep Med. (2007) 8:651–5. 10.1016/j.sleep.2006.11.013 17420154

[136] Lockley SW Dijk DJ Kosti O Skene DJ Arendt J. Alertness, mood and performance rhythm disturbances associated with circadian sleep disorders in the blind. J Sleep Res. (2008) 17:207–16. 10.1111/j.1365-2869.2008.00656.x 18482109 PMC7611877

[137] Lewy AJ Bauer VK Hasler BP Kendall AR Pires ML Sack RL. Capturing the circadian rhythms of free-running blind people with 0.5 mg melatonin. Brain Res. (2001) 918:96–100. 10.1016/S0006-8993(01)02964-X 11684046

[138] Roth T Nir T Zisapel N. Prolonged release melatonin for improving sleep in totally blind subjects: a pilot placebo-controlled multicenter trial. Nat Sci Sleep. (2015) 7:13–23. 10.2147/NSS.S71838 25678831 PMC4319556

[139] Warman GR Pawley MD Bolton C Cheeseman JF Fernando AT Arendt J. Circadian-related sleep disorders and sleep medication use in the New Zealand blind population: an observational prevalence survey. PLoS ONE. (2011) 6:e22073. 10.1371/journal.pone.0022073 21789214 PMC3138759

[140] Flynn-Evans EE Shekleton JA Miller B Epstein LJ Kirsch D Brogna LA. Circadian phase and phase angle disorders in primary insomnia. Sleep. (2017) 40:zsx163. 10.1093/sleep/zsx163 29029340

[141] Wittmann M Dinich J Merrow M Roenneberg T. Social jetlag: misalignment of biological and social time. Chronobiol Int. (2006) 23:497–509. 10.1080/07420520500545979 16687322

[142] Fu L Lee CC. The circadian clock: pacemaker and tumour suppressor. Nat Rev Cancer. (2003) 3:350–61. 10.1038/nrc1072 12724733

[143] Filipski E Delaunay F King VM Wu MW Claustrat B Grechez-Cassiau A. Effects of chronic jet lag on tumor progression in mice. Cancer Res. (2004) 64:7879–85. 10.1158/0008-5472.CAN-04-0674 15520194

[144] Costa G Haus E Stevens R. Shift work and cancer - considerations on rationale, mechanisms, and epidemiology. Scand J Work Environ Health. (2010) 36:163–79. 10.5271/sjweh.2899 20126969

[145] Lapin V. Pineal influences on tumor. Prog Brain Res. (1979) 52:523–33. 10.1016/S0079-6123(08)62960-X 232927

[146] Tamarkin L Cohen M Roselle D Reichert C Lippman M Chabner B. Melatonin inhibition and pinealectomy enhancement of 7,12-dimethylbenz(a)anthracene-induced mammary tumors in the rat. Cancer Res. (1981) 41(11 Pt 1):4432–6. 6796259

[147] Marie Hansen A Helene Garde A Hansen J. Diurnal urinary 6-sulfatoxymelatonin levels among healthy danish nurses during work and leisure time. Chronobiol Int. (2006) 23:1203–15. 10.1080/07420520601100955 17190706

[148] Stevens RG Davis S. The melatonin hypothesis: electric power and breast cancer. Environ. Health Perspect. (1996) 104(Suppl. 1):135–40. 10.1289/ehp.96104s1135 8722117 PMC1469562

[149] Schernhammer ES. RE: night shift work and breast cancer incidence: three prospective studies and meta-analysis of published studies. J. Natl. Cancer Inst. (2017) 109:djw169. 10.1093/jnci/djx002 28376171

[150] Hansen J. Night shift work and risk of breast cancer. Curr Environ Health Rep. (2017) 4:325–39. 10.1007/s40572-017-0155-y 28770538

[151] Blask DE Sauer LA Dauchy RT. Melatonin as a chronobiotic/anticancer agent: cellular, biochemical, and molecular mechanisms of action and their implications for circadian-based cancer therapy. Curr Top Med Chem. (2002) 2:113–32. 10.2174/1568026023394407 11899096

[152] Reiter RJ Rosales-Corral SA Tan DX Acuna-Castroviejo D Qin L Yang SF. (2017). Melatonin, a full service anti-cancer agent: inhibition of initiation, progression and metastasis. Int. J. Mol. Sci. 18:e843. 10.3390/ijms18040843 28420185 PMC5412427

[153] Blask DE Brainard GC Dauchy RT Hanifin JP Davidson LK Krause JA. Melatonin-depleted blood from premenopausal women exposed to light at night stimulates growth of human breast cancer xenografts in nude rats. Cancer Res. (2005) 65:11174–84. 10.1158/0008-5472.CAN-05-1945 16322268

[154] Hill SM Blask DE Xiang S Yuan L Mao L Dauchy RT. Melatonin and associated signaling pathways that control normal breast epithelium and breast cancer. J Mammary Gland Biol Neoplasia. (2011) 16:235–45. 10.1007/s10911-011-9222-4 21773809

[155] Blask DE Dauchy RT Dauchy EM Mao L Hill SM Greene MW. Light exposure at night disrupts host/cancer circadian regulatory dynamics: impact on the Warburg effect, lipid signaling and tumor growth prevention. PLoS ONE. (2014) 9:e102776. 10.1371/journal.pone.0102776 25099274 PMC4123875

[156] Hill SM Belancio VP Dauchy RT Xiang S Brimer S Mao L. Melatonin: an inhibitor of breast cancer. Endocr Relat Cancer. (2015) 22:R183–204. 10.1530/ERC-15-0030 25876649 PMC4457700

[157] Lissoni P Chilelli M Villa S Cerizza L Tancini G Five years survival in metastatic non-small cell lung cancer patients treated with chemotherapy alone or chemotherapy and melatonin: a randomized trial. J Pineal Res. (2003) 35:12–5. 10.1034/j.1600-079X.2003.00032.x 12823608

[158] Touitou Y Haus E Biologic Rhythms in Clinical and Laboratory Medicine. Heidelberg, Springer-Verlag (1992).

[159] Morgan L Hampton S Gibbs M Arendt J. Circadian aspects of postprandial metabolism. Chronobiol. Int. (2003) 20:795–808. 10.1081/CBI-120024218 14535354

[160] Scheer FAJL Hilton MF Mantzoros CS Shea SA. Adverse metabolic and cardiovascular consequences of circadian misalignment. Proc Natl Acad Sci USA. (2009) 106:4453–8. 10.1073/pnas.0808180106 19255424 PMC2657421

[161] Stenvers DJ Scheer FAJL Schrauwen P la Fleur SE Kalsbeek A. Circadian clocks and insulin resistance. Nat. Rev. Endocrinol. (2019) 15:75–89. 10.1038/s41574-018-0122-1 30531917

[162] Ang JE Revell V Mann A Mäntele S Otway DT Johnston JD. Identification of human plasma metabolites exhibiting time-of-day variation using an untargeted liquid chromatography-mass spectrometry metabolomic approach. Chronobiol Int. (2012) 29:868–81. 10.3109/07420528.2012.699122 22823870 PMC3433180

[163] Davies SK Ang JE Revell VL Holmes B Mann A Robertson FP. Effect of sleep deprivation on the human metabolome. Proc Natl Acad Sci USA. (2014) 111:10761–6. 10.1073/pnas.1402663111 25002497 PMC4115565

[164] Giskeødegård GF Davies SK Revell VL Keun H Skene DJ. Diurnal rhythms in the human urine metabolome during sleep and total sleep deprivation. Sci Rep. (2015) 5:14843. 10.1038/srep14843 26450397 PMC4598809

[165] Goyal A Terry PD Superak HM Nell-Dybdahl CL Chowdhury R Phillips LS. Melatonin supplementation to treat the metabolic syndrome: a randomized controlled trial. Diabetol Metab Syndr. (2014) 6:124. 10.1186/1758-5996-6-124 25937837 PMC4416300

[166] Van Cauter E Blackman JD Roland D Spire JP Refetoff S Polonsky KS. Modulation of glucose regulation and insulin secretion by circadian rhythmicity and sleep. J Clin Invest. (1991) 88:934–42. 10.1172/JCI115396 1885778 PMC295490

[167] Morgan L Arendt J Owens D Folkard S Hampton S Deacon S. Effects of the endogenous clock and sleep time on melatonin, insulin, glucose and lipid metabolism. J Endocrinol. (1998) 157:443–51. 10.1677/joe.0.1570443 9691977

[168] Johnston JD. Physiological responses to food intake throughout the day. Nutr Res Rev. (2014) 27:107–18. 10.1017/S0954422414000055 24666537 PMC4078443

[169] Lund J Arendt J Hampton SM English J Morgan LM. Postprandial hormone and metabolic responses amongst shift workers in Antarctica. J Endocrinol. (2001) 171:557–64. 10.1677/joe.0.1710557 11739022

[170] la Fleur SE Kalsbeek A Wortel J van der Vliet J Buijs RM. Role for the pineal and melatonin in glucose homeostasis: pinealectomy increases night-time glucose concentrations. J. Neuroendocrinol. (2001) 13:1025–32. 10.1046/j.1365-2826.2001.00717.x 11722698

[171] Owino S Contreras-Alcantara S Baba K Tosini G. Melatonin signaling controls the daily rhythm in blood glucose levels independent of peripheral clocks. PLoS ONE. (2016) 11:e0148214. 10.1371/journal.pone.0148214 26824606 PMC4732609

[172] Rubio-Sastre P Scheer FA Gómez-Abellán P Madrid JA Garaulet M. Acute melatonin administration in humans impairs glucose tolerance in both the morning and evening. Sleep. (2014) 37:1715–9. 10.5665/sleep.4088 25197811 PMC4173928

[173] Garaulet M Gómez-Abellán P Rubio-Sastre P Madrid JA Saxena R Scheer FA. Common type 2 diabetes risk variant in MTNR1B worsens the deleterious effect of melatonin on glucose tolerance in humans. Metab Clin Exp. (2015) 64:1650–7. 10.1016/j.metabol.2015.08.003 26440713 PMC4856010

[174] Peschke E Bähr I Mühlbauer E. Melatonin and pancreatic islets: interrelationships between melatonin, insulin and glucagon. Int J Mol Sci. (2013) 14:6981–7015. 10.3390/ijms14046981 23535335 PMC3645673

[175] West RJ Lloyd JK Turner WM. Familial insulin-resistant diabetes, multiple somatic anomalies, and pineal hyperplasia. Arch Dis Child. (1975) 50:703–8. 10.1136/adc.50.9.703 1190820 PMC1545638

[176] Bathi RJ Parveen S Mutalik S Rao R. Rabson-mendenhall syndrome: two case reports and a brief review of the literature. Odontology. (2010) 98:89–96. 10.1007/s10266-009-0106-7 20155514

[177] Bouatia-Naji N Bonnefond A Cavalcanti-Proença C Sparsø T Holmkvist J Marchand M. A variant near MTNR1B is associated with increased fasting plasma glucose levels and type 2 diabetes risk. Nat. Genet. (2009) 41:89–94. 10.1038/ng.277 19060909

[178] Bonnefond A Clément N Fawcett K Yengo L Vaillant E Guillaume JL. Rare MTNR1B variants impairing melatonin receptor 1B function contribute to type 2 diabetes. Nat Genet. (2012) 44:297–301. 10.1038/ng.1053 22286214 PMC3773908

[179] Spiegel K Tasali E Leproult R Van Cauter E. Effects of poor and short sleep on glucose metabolism and obesity risk. Nat Rev Endocrinol. (2009) 5:253–61. 10.1038/nrendo.2009.23 19444258 PMC4457292

[180] Van Cauter E. Sleep disturbances and insulin resistance. Diabet Med. (2011) 28:1455–62. 10.1111/j.1464-5491.2011.03459.x 21950773

[181] Bonnefond A Froguel P. Disentangling the role of melatonin and its receptor MTNR1B in type 2 diabetes: still a long way to go? Curr Diab Rep. (2017) 17:122. 10.1007/s11892-017-0957-1 29063374

[182] Scheer FA Kalsbeek A Buijs RM. Cardiovascular control by the suprachiasmatic nucleus: neural and neuroendocrine mechanisms in human and rat. Biol Chem. (2003) 384:697–709. 10.1515/BC.2003.078 12817466

[183] Lemmer B. The importance of circadian rhythms on drug response in hypertension and coronary heart disease–from mice and man. Pharmacol Ther. (2006) 111:629–51. 10.1016/j.pharmthera.2005.11.008 16480770

[184] Lemmer B. Chronopharmacology and controlled drug release. Expert Opin Drug Deliv. (2005) 2:667–81. 10.1517/17425247.2.4.667 16296793

[185] Sun H Gusdon AM Qu S. Effects of melatonin on cardiovascular diseases: progress in the past year. Curr Opin Lipidol. (2016) 27:408–13. 10.1097/MOL.0000000000000314 27075419 PMC4947538

[186] Scheer FA Van Montfrans GA van Someren EJ Mairuhu G Buijs RM. Daily nighttime melatonin reduces blood pressure in male patients with essential hypertension. Hypertension. (2004) 43:192–7. 10.1161/01.HYP.0000113293.15186.3b 14732734

[187] Scheer FA. Potential use of melatonin as adjunct antihypertensive therapy. Am J Hypertens. (2005) 18(12 Pt 1):1619–20. 10.1016/j.amjhyper.2005.07.013 16364835

[188] Macleod MR O'Collins T Horky LL Howells DW Donnan GA. Systematic review and meta-analysis of the efficacy of melatonin in experimental stroke. J Pineal Res. (2005) 38:35–41. 10.1111/j.1600-079X.2004.00172.x 15617535

[189] O'Collins VE Macleod MR Cox SF Van Raay L Aleksoska E Donnan GA. Preclinical drug evaluation for combination therapy in acute stroke using systematic review, meta-analysis, and subsequent experimental testing. J Cereb Blood Flow Metab. (2011) 31:962–75. 10.1038/jcbfm.2010.184 20978519 PMC3063631

[190] Wilkinson M Arendt J Bradtke J de Ziegler D Determination of dark- induced elevation of pineal N-acetyl-transferase with simultaneous radioimmunoassay of melatonin in pineal, serum and pituitary of the male rat. J Endocrinol. (1977) 72:243–4. 10.1677/joe.0.0720243 845539

[191] Middleton B. Measurement of melatonin and 6-sulphatoxymelatonin. Methods Mol Biol. (2013) 1065:171–99. 10.1007/978-1-62703-616-0_11 23996364

[192] Arendt J Bojkowski C Franey C Wright J Marks V. Immunoassay of 6-hydroxymelatonin sulfate in human plasma and urine: abolition of the urinary 24-hour rhythm with atenolol. J Clin Endocrinol Metab. (1985) 60:1166–73. 10.1210/jcem-60-6-1166 3998065

[193] Naidoo R Investigation of Rhythmic Endocrine Function in Intensive Care With Emphasis on Melatonin. Doctoral Thesis, University of Surrey, Surrey, United Kingdom (1999).

[194] Arendt J. Melatonin: characteristics, concerns, and prospects. J Biol Rhythms. (2005) 20:291–303. 10.1177/0748730405277492 16077149

[195] Arendt J Mammalian pineal rhythms. Pineal Res Rev. (1985) 3:161–213.

[196] Lewy AJ Sack RL. The dim light melatonin onset as a marker for circadian phase position. Chronobiol Int. (1989) 6:93–102. 10.3109/07420528909059144 2706705

[197] Revell VL Arendt J Terman M Skene DJ. Short-wavelength sensitivity of the human circadian system to phase-advancing light. J Biol Rhythms. (2005) 20:270–2. 10.1177/0748730405275655 15851533

[198] Gibbs M Hampton S Morgan L Arendt J. Adaptation of the circadian rhythm of 6-sulphatoxymelatonin to a shift schedule of seven nights followed by seven days in offshore oil installation workers. Neurosci Lett. (2002) 325:91–4. 10.1016/S0304-3940(02)00247-1 12044629

[199] Gibbs M Hampton S Morgan L Arendt J. Predicting circadian response to abrupt phase shift: 6-sulphatoxymelatonin rhythms in rotating shift workers offshore. J Biol Rhythms. (2007) 22:368–70. 10.1177/0748730407302843 17660453

[200] Arendt J Middleton B Williams P Francis G Luke C. Sleep and circadian phase in a ship's crew. J Biol Rhythms. (2006) 21:214–21. 10.1177/0748730405285278 16731661

[201] Lévi F Focan C Karaboué A de la Valette V Focan-Henrard D Baron B. Implications of circadian clocks for the rhythmic delivery of cancer therapeutics. Adv. Drug Deliv. Rev. (2007) 59:1015–35. 10.1016/j.addr.2006.11.001 17692427

